# Assay of Ds2 Transposition Activity in the Maize W22

**DOI:** 10.17912/micropub.biology.001902

**Published:** 2026-02-02

**Authors:** Harpreet Kaur, Matthew Bacchus, Lilly Horowitz, Caroline Larow, Vedant Rawat, Dafang Wang

**Affiliations:** 1 Biology, Hofstra University, Hempstead, NY, US

## Abstract

*Ds* elements are non-autonomous DNA transposons requiring *Ac* transposase for mobility. From the 39 *Ds2* elements reported in B73, we identified 38 *Ds2* homologous elements in the W22, 30 of which retained intact terminal inverted repeats and target site duplications. PCR-based excision assays on 19 loci in embryos and leaves revealed the majority are immobile; only *Ds2-23* showed suggestive somatic transposition. Targeted bisulfite sequencing revealed that while *Ac* reduces methylation, absolute levels remain stable and do not correspond to the varied transposition activities. This suggests structural integrity or DNA methylation alone does not predict *Ds2* transposition variation in W22.

**Figure 1. Transposition of Ds2 elements and DNA methylation profiles.**
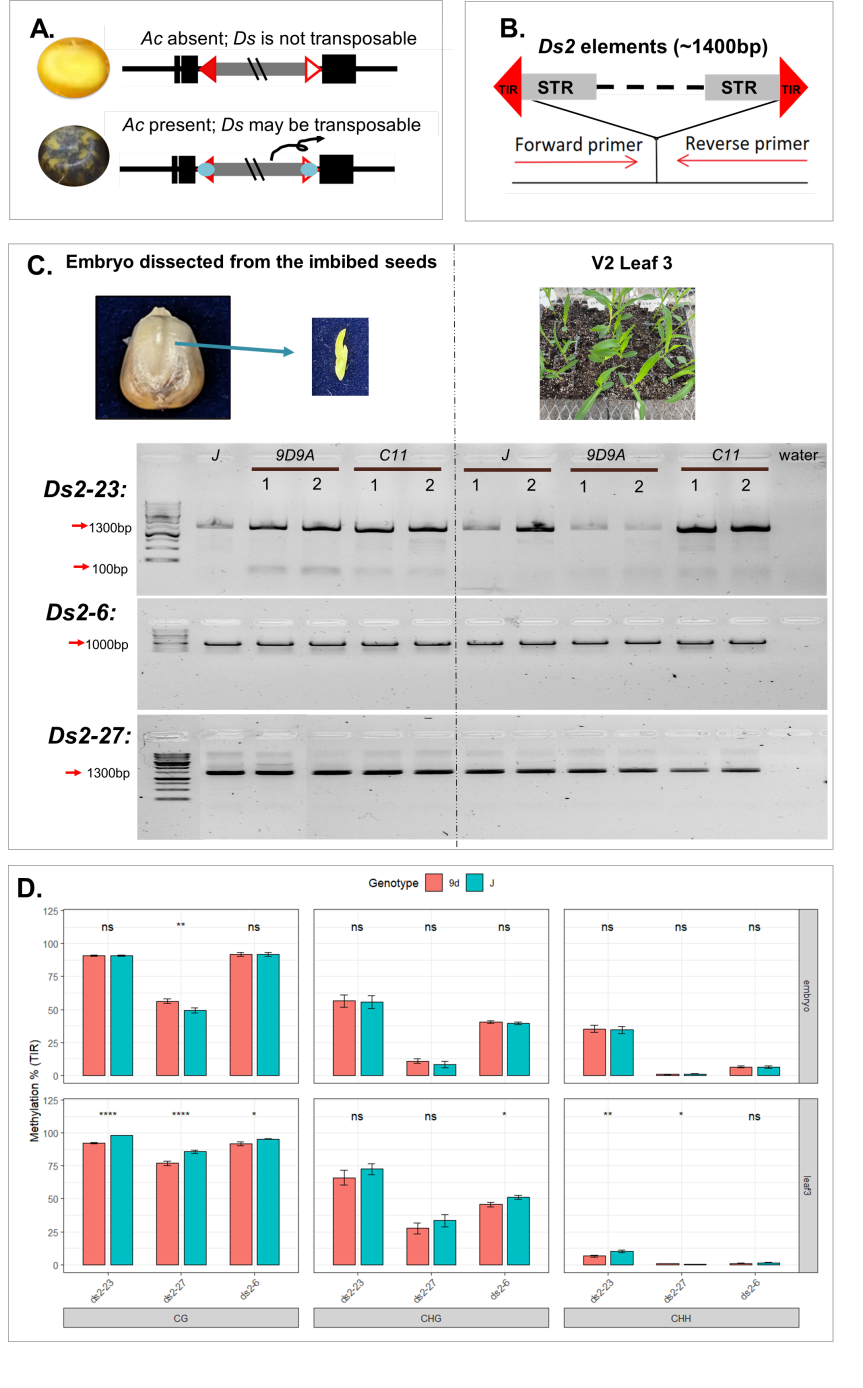
**A.** Reporter system for *Ac* activity. Maize lines without *Ac* (left) exhibit seeds with no spots due to the disruption of the *R1* gene ( *r1-m3* allele) by a *Ds* insertion. Maize lines with *Ac* (right) exhibit seeds with coarse purple spots resulting from the somatic excision of the tester *Ds* from the *R1* locus ( *r1-m3* allele) in the presence of *Ac* transposase. Black boxes represent *R1* exons, with exon 2 disrupted by the *Ds* element. Grey lines with red triangles indicate the *Ds* elements inserted into *R1* exon 2. **B.** The design of PCR assays for detecting *Ds2* excision. *Ds2* elements are characterized by intact terminal inverted repeats (TIRs) and subterminal repeat sequences (STRs) flanking variable internal sequences. Primers were uniquely designed from the flanking sequences at each specific *Ds2* locus. **C.** Somatic transposition activity of *Ds2-23* , *Ds2-27* , and *Ds2-6* in embryo and leaf tissues. The top panel displays embryos dissected from seeds 41 hours post-imbibition and the third leaf at the V2 developmental stage. Gel images show PCR products generated using genome-specific primers. Genotypes include J ( *r1-m3* allele, *Ac* -absent); 9D9A and C11 ( *r1-m3* allele, *Ac* present at the *p1* gene); and W22 (wild-type inbred line lacking both the *r1-m3* allele and active *Ac* ). Numbers 1-2 indicate biological replicates. Red arrows with labeled band sizes indicate expected products for either the *Ds* insertion or the excision footprint. PCR band sizes varied according to the individual lengths of *Ds2* (Supplemental Data 1). **D.** Targeted bisulfite sequencing of TIR/STR regions. Bisulfite sequencing was performed on *Ds2-23* , *Ds2-27* , and *Ds2-6* to quantify methylation levels in the presence or absence of *Ac* . Sequencing coverage for each locus spanned the flanking genomic DNA and the first 250 bp of the *Ds2* element. Specific primer sequences are provided in the Methods section. Data represents the average of two biological replicates per locus. Sample sizes for each context are as follows: CG sites: N=46 (23 sites from two biological replicates) across all three elements; CHG sites: N=16 (8 sites) for *Ds2-23* , N=24 (12 sites) for *Ds2-27* , and N=8 (4 sites) for *Ds2-6* ; CHH sites: N=18 (9 sites) for *Ds2-23* , N=14 (7 sites) for *Ds2-27* , and N=12 (6 sites) for *Ds2-6* .

## Description

Transposable elements (TEs) are mobile genetic sequences first identified in maize by Barbara McClintock (McClintock, 1950; 1951; 1956). In maize, TEs account for approximately 85% of the genomic DNA (Schnable et al, 2012). The *Activator/Dissociation* ( *Ac/Ds* ) family includes the autonomous *Ac* element, which encodes a transposase that catalyzes the excision and reintegration of non-autonomous *Ds* elements. *Ds* elements arise from *Ac* through internal deletions or substitutions (Fedoroff et al, 1983; Conrad et al, 2007; Vollbrecht et al, 2010; Lazarow et al, 2013). Genome-wide analysis of the B73 reference genome previously identified nearly 900 *Ds* and *Ds* -like elements, classified into four subfamilies: *Ds1* , *Ds2* , *Ds3L* , and *Ds4L* (Du et al, 2011).

*Ds* transposition is driven by the *Ac* transposase. The terminal inverted repeats (TIRs) and subterminal repeats (STRs) of *Ds* are conserved sequences essential for transposase recognition and binding (Kunze et al, 1989). Cytosine methylation, particularly within GC-rich STRs, has been implicated in regulating *Ds* activity (Len et al 1992; Ott et al, 1992; Brutnell, et al, 1994; Wang et al, 1996; 1998). Among the subfamilies, *Ds1* and *Ds2* are predicted to retain mobility due to conserved STR motifs harboring *Ac* -binding sites (Du et al, 2011).

Here, we focused on 39 *Ds2* elements annotated in B73 that retain intact STR sequences and possess the potential to transpose. We examined these elements in the W22 maize background, a line historically used for studying *Ac/Ds* and *Mutator* ( *Mu* ) families (Lisch, 2009; Jiang, et al, 2019). The objectives of this study were to: (i) identify W22 homologous elements of B73 *Ds2* elements, (ii) assess their transposition capacity in embryos and young leaves, and (iii) evaluate whether variation in DNA methylation within STR regions is associated with the presence of *Ac* and transposition activity.

We performed BLAST searches to identify homologous elements in the W22 inbred line corresponding to the 39 *Ds2* elements previously annotated in the B73 reference genome (Schnable et al, 2012; Springer, et al, 2018). Of these, 38 homologous elements were recovered. Thirty of these elements retained the characteristic TIRs and were flanked by TSDs (Supplemental Data 1). These 30 characteristic elements in W22 shared high sequence similarity with their B73 counterparts (>90%), with length differences ranging from 0% to 45% (Supplemental Data 1). These elements represent only the W22 homologous elements of B73 *Ds2* ; W22-specific *Ds2* elements were not included, as a genome-wide annotation was not conducted in this study.

To assess *Ds2* mobility, we utilized two classes of genotypes: *Ac* -present lines ( *p1-vv9D9A* and *p1-wwC11* ) and *Ac* -absent controls (J and W22). All lines except W22 (specifically *p1-vv9D9A* , *p1-wwC11* , and J) carry the *r1-m3* allele, a specialized "tester" for *Ac* activity containing a *Ds* insertion at the *R1* locus (Kermicle, 1970; Federoff et al., 1983). To ensure genomic consistency, these tester stocks were backcrossed to the W22 over multiple generations. Despite the predominantly W22 background, residual donor DNA from the tester lines likely persists, explaining the observed sequence heterogeneity in specific genomic regions. In the *Ac* -present lines, the *Ds* element was excised from the *R1* gene by the *Ac* transposase, restoring anthocyanin pigmentation and producing purple spots in the aleurone layer of the endosperm (Figure A), which is to confirm that the *Ac* transposase was active and competent to catalyze *Ds* excision at independent loci within the W22 background.

For each *Ds2* element tested in W22, primers were designed from the ±500 bp flanking sequences (Figure B). In *Ac* - backgrounds (W22, J), where *Ds2* elements are immobile, PCR amplification produced only the expected large insertion bands. In *Ac* + lines, transposition was expected to generate smaller bands following excision. We tested germinating embryos and young leaves (V2 stage), representing early developmental stages where *Ac/Ds* transposition is typically active.&nbsp;

Of the 38 homologous elements identified, 31 were selected for testing, including 27 with intact TIRs/TSDs and four lacking one or both features. PCR amplification yielded the expected insertion bands for 19 of these elements. Among them, only *Ds2-23* generated smaller fragments consistent with somatic excision. These putative excision products were consistently observed across biological replicates in *Ac+* backgrounds but were absent in *Ac–* controls in both embryonic and leaf tissues (Figure C). While these results suggest somatic excision of *Ds2-23* , low band intensity prevented the recovery of sufficient PCR product for Sanger sequencing. Consequently, the "active" status of *Ds2-23* remains tentative pending future cloning experiments to confirm the excision footprint sequences. The remaining 18 elements showed no observable bands corresponding to excision footprints; therefore, we conclude they are either immobile or possess negligible transposition activity within the tested tissues.

To assess whether cytosine methylation correlates with transposition potential, we performed targeted bisulfite sequencing of three *Ds2* elements: the potentially active *Ds2-23* and two inactive elements ( *Ds2-6* and *Ds2-27* ) (Supplementary Data 2). Methylation in CG, CHG, and CHH contexts was quantified across the first 250 bp of each element, including sub-terminal regions (STRs) expected to be methylation targets (Kunze et al., 1989; Len et al 1992; Ott et al, 1992; Brutnell, et al, 1994; Wang et al, 1996; 1998). This region includes a varying number of cytosine sites (e.g., 23 CG sites for all the tested Ds2; 8 CHG sites in *Ds2-23* ; 12 in *Ds2-27* ; and 4 in *Ds2-6;* 9 CHH sites in *Ds2-23* ; 7 in *Ds2-27* ; and 6 in *Ds2-6* ).&nbsp;

The average DNA methylation levels and standard errors are presented in Figure D. In the embryo, the presence of *Ac* did not significantly alter cytosine methylation levels, except for *Ds2-27* . In leaves, methylation significantly decreased in the presence of *Ac* in the CG and CHH contexts for *Ds2-23* and *Ds2-27* , and in the CG and CHG contexts for *Ds2-6* . However, despite these statistically significant reductions, the absolute DNA methylation levels remained notably stable across all genotypes and do not induce transposition activities in *Ds2-27* and *Ds2-6* . This observation mirrors the behavior of the cryptic *Ac* -homologous sequence, *cAc-11* , which shares over 90% homology with known *Ac* sequences and possesses the characteristic 11-bp terminal inverted repeats and 8-bp target site duplications of the *Ac/Ds* family (Len et al, 1992). In that system, the presence of *Ac* failed to eliminate hypermethylation or recover the activity of the cryptic element. However, that study lacked quantitative analysis to determine whether methylation was also significantly reduced yet remained above a functional threshold, as observed in our results. Interestingly, while the potentially mobile *Ds2-23* also showed a decrease in DNA methylation, its absolute methylation levels remained higher than those of the inactive *Ds2-27* and *Ds2-6* . This suggests that factors beyond DNA methylation, such as local chromatin environment or position effects, may be involved in the immobilization of *Ds2-27* and *Ds2-6* or the activation of *Ds2-23* .

Our results demonstrate that most *Ds2* elements in B73 have homologous elements in W22 with intact terminal inverted repeats (TIRs) and target site duplications (TSDs). Despite this structural integrity, the W22 genome maintains stable silencing across most *Ds2* loci, likely through DNA methylation. While the presence of *Ac* induces statistically significant reductions in methylation, these shifts appear biologically insufficient to restore mobility. Of 19 loci tested, only *Ds2-23* showed evidence of somatic transposition. Notably, this activity did not correlate with lower methylation levels compared to other lines. Consequently, neither structural integrity nor DNA methylation levels serve as reliable predictors of *Ds2* transposition, suggesting that regulation is likely enforced by additional mechanisms such as the local chromatin environment or position effects.

## Methods

**Genome Searches** Sequences and genomic locations of all *Ds2* elements were obtained from Du et al. using the B73 reference genome. Because the *Ac* lines used in this study were originally derived from the W22 background, we performed BLAST searches of *Ds2* elements in the W22 genome to obtain their sequences along with 500 bp of upstream and downstream flanking regions. Primers were designed from these flanking regions using the NCBI Primer-BLAST tool. Three primer pairs were designed and tested for each *Ds2* element.

**Plant Growth** Maize lines p1-vv9D9A, p1-wwC11, and the wild-type line J were obtained from Dr. Thomas Peterson’s laboratory at Iowa State University. The W22 line was obtained from the Maize Genetics Cooperation Stock Center. Seeds were germinated in Petri dishes containing two sheets of paper towel moistened with 3 mL of water, followed by 500 µL of water every 24 h. Germination was carried out at 28 °C in an incubator, and embryos were dissected from the imbibed seeds after 41 h of incubation. For whole-plant growth, seeds were germinated in small pots containing Premier B10281RG ProMix in a greenhouse maintained at 25 °C. The entire third leaf was collected at the V2 stage.

**DNA Extraction** Genomic DNA was extracted from 41-hour imbibed embryos and from third leaves at the V2 stage. Two to three independent plants of the same genotype were processed using the Quick-DNA™ Plant/Seed Miniprep Kit (Zymo Research, D6020) following the manufacturer’s protocol. DNA quality was assessed using *ZmTUB* (Zm00001d010275) primers, and all samples were confirmed to be PCR-amplifiable.

**PCR** Somatic transpositions in lines carrying active *Ac* were detected by PCR using PCR Master Mix (Sydlabs, MB067-EQ2B). Each reaction contained primers specific to the *Ds2* flanking sequence (primer sequences listed in “Reagents”), with nuclease-free water serving as a negative control. PCR amplification conditions were: initial denaturation at 95 °C for 30 s; 35 cycles of 95 °C for 30 s, 55 °C for 30 s, 72 °C for 30 s; and a final extension at 72 °C for 5 min. Amplified products were separated on 1% agarose gels and visualized using the GelDoc Go Imaging System (Bio-Rad).

**Targeted Bisulfite Sequencing** For each genotype and tissue type, genomic DNA from two independent biological replicates was sent to CD Genomics (Shirley, NY) for library preparation and sequencing (primer sequences listed in “Reagents”). Primers targeting the subterminal regions (1–250 bp) of the TIR and STR sequences were used. Methylation data were averaged across replicates for presentation in the figure.

## Reagents

**Table:** 

**Targeted Bisulfite Sequencing**		
Ds2-6_473-720 (Ds2-6 subterminal 1-220bp)	AAATGTTGTTTAGTATTATTTGTTATTTTTAGATTTAGG	ATTTCATAATATAATTTTACCRAACAAAAATACC
*Ds2-23_450-707* (Ds2-23 subterminal 1-207bp)	GAGGAGTTATGGGTTGTGGAGGTTGT	RCCTACTCTCTCCCTATCTCTCAACC
*Ds2-27_501-770* (Ds2-27 subterminal 1-270bp)	TGTATAATTAGGGATGAAAGTAGGATG	ATCTAAATTACAACAAACTATATTAAAATAATCCC
